# Roles of Anxiety and Depression in Predicting Cardiovascular Disease Among Patients With Type 2 Diabetes Mellitus: A Machine Learning Approach

**DOI:** 10.3389/fpsyg.2021.645418

**Published:** 2021-04-28

**Authors:** Haiyun Chu, Lu Chen, Xiuxian Yang, Xiaohui Qiu, Zhengxue Qiao, Xuejia Song, Erying Zhao, Jiawei Zhou, Wenxin Zhang, Anam Mehmood, Hui Pan, Yanjie Yang

**Affiliations:** ^1^Department of Medical Psychology, Harbin Medical University, Harbin, China; ^2^Department of Endocrinology, Peking Union Medical College Hospital, Beijing, China

**Keywords:** type 2 diabetes mellitus, cardiovascular disease, bio-psycho-social factors, machine learning, China

## Abstract

Cardiovascular disease (CVD) is a major complication of type 2 diabetes mellitus (T2DM). In addition to traditional risk factors, psychological determinants play an important role in CVD risk. This study applied Deep Neural Network (DNN) to develop a CVD risk prediction model and explored the bio-psycho-social contributors to the CVD risk among patients with T2DM. From 2017 to 2020, 834 patients with T2DM were recruited from the Department of Endocrinology, Affiliated Hospital of Harbin Medical University, China. In this cross-sectional study, the patients' bio-psycho-social information was collected through clinical examinations and questionnaires. The dataset was randomly split into a 75% train set and a 25% test set. DNN was implemented at the best performance on the train set and applied on the test set. The receiver operating characteristic curve (ROC) analysis was used to evaluate the model performance. Of participants, 272 (32.6%) were diagnosed with CVD. The developed ensemble model for CVD risk achieved an area under curve score of 0.91, accuracy of 87.50%, sensitivity of 88.06%, and specificity of 87.23%. Among patients with T2DM, the top five predictors in the CVD risk model were body mass index, anxiety, depression, total cholesterol, and systolic blood pressure. In summary, machine learning models can provide an automated identification mechanism for patients at CVD risk. Integrated treatment measures should be taken in health management, including clinical care, mental health improvement, and health behavior promotion.

## Introduction

Diabetes Mellitus (DM) is a complex metabolic disorder characterized by chronic high glucose levels, insulin resistance, and declining insulin secretion from the pancreas (American Diabetes Association, 2014). According to the International Diabetes Federation (Saeedi et al., 2019), there are 463 million people with diabetes worldwide, and this number will increase to 700 million by 2045. Cardiovascular disease (CVD) is the leading cause of disability and death in patients with DM, accounting for 70–80% (Kibel et al., 2017). Patients with DM are 2–4 times more likely to suffer from coronary heart disease, myocardial infarction, angina pectoris, etc., than those without DM (Larsson et al., 2018; American Diabetes Association, 2019). The global costs of diabetes are large and will substantially increase from $1.3 trillion in 2015 to $2.1-$2.5 trillion by 2030 (Bommer et al., 2018). Type 2 diabetes mellitus (T2DM) is the most prevalent form of diabetes affecting over 90% of diabetic population worldwide (Zheng et al., 2018). CVD costs contributed 20–49% of the total direct costs of treating T2DM (Einarson et al., 2018). Given the substantial clinical and economic burden of CVD in people with T2DM, it is of profound significance to develop a prediction model of cardiovascular complications among Chinese patients with T2DM.

Guidelines for the management of T2DM advocate calculating CVD risk to guide the initiation of appropriate treatment. The Framingham heart study, a transgenerational cohort study, determined the individual 10-year risk of developing CVD by evaluating the age, sex, smoking status, blood pressure, total cholesterol (TC) level, high-density lipoprotein cholesterol (HDL-C) level, low-density lipoprotein cholesterol (LDL-C) level, and other information (Mahmood et al., 2014). Metabolic syndrome is also a useful tool for the prevention of CVD, which has defined a cluster of conditions including waist-based obesity, hypertension, hyperglycemia, and abnormal cholesterol or triglyceride levels (Lee et al., 2019). International Diabetes Federation recommends using the United Kingdom Prospective Diabetes Study risk engine, which is dedicated to the T2DM population (Kothari et al., 2002). Early detection and prompt treatment may be able to prevent or delay troubling cardiovascular complications and the related economic costs.

In recent years, with ever-growing data from hospitals, there are great benefits of employing machine learning technology to provide insights, augment prevention, and reduce costs in healthcare settings. Multiple machine learning models have been applied for producing the risk of developing complications in diabetic patients (Zarkogianni et al., 2018). Nowak et al. developed a cardiovascular risk prediction system using gradient-boosted machine learning and lasso regularized Cox regression and revealed that multiprotein arrays could be useful in identifying individuals with T2DM who were at the highest risk of CVD (Nowak et al., 2018). Baum et al. examined the impact of weight loss on reducing cardiovascular complications of T2DM using a causal forest modeling and suggested that the prediction model had high accuracy (Baum et al., 2017).

However, most existing prediction models focus on the biological indicators and several socio-demographic characteristics, ignoring the predictive role of psychological determinants. The prevalence of CVD is due to a combination of biological and psycho-social factors. These factors include age, sex, smoking, drinking, overweight and obesity, high-fat and high-cholesterol diet, insulin resistance, hyperglycemia, hypertension, lipid metabolism disorders, mental disorders (Stewart et al., 2017; van der Ende et al., 2017; Petrie et al., 2018; Ricci et al., 2018). Particularly, researches over the last 10 years have reported the predictive roles of depression and anxiety in the onset and progression of cardiovascular outcomes (Hiriscau and Bodolea, 2019; Meyer et al., 2019; Caruso et al., 2020). Among patients with T2DM, Hazuda et al. carried out a median follow-up of 9.6 years study and revealed that depression contributed to the composite secondary CVD outcomes (Hazuda et al., 2019). From Evaluation of Diabetes Treatment Study, Deschênes et al. identified four distinct trajectories of anxiety and reported that anxiety was significantly associated with an increased risk of CVD among patients with T2DM (Deschênes et al., 2018). Although healthcare workers and scholars have confirmed the important role of mental health in preventing CVD among patients with T2DM, existing CVD prediction models lack relevant factors, especially anxiety and depression. Therefore, this study applied Deep Neural Network (DNN) to develop a CVD risk prediction model and explored the bio-psycho-social contributors to the CVD risk in patients with T2DM.

## Materials and Methods

### Sample

This study was conducted in Harbin, Heilongjiang province, China, from 2017 to 2020. A total of 834 patients with T2DM were recruited from the Department of Endocrinology, Affiliated Hospital of Harbin Medical University. Patients eligible for inclusion in this study were adults with T2DM and voluntary participation. Exclusion criteria included type 1 diabetes mellitus, voluntary problems, and severe diseases affecting safety. In this study, coronary heart disease, peripheral vascular disease, myocardial infarction, and heart failure events were considered as cardiovascular complications of T2DM.

### Ethical Approval

This study protocol was approved by the Ethics Committee of Harbin Medical University. Patients or their guardians were informed of the information of this study, and informed consent was obtained from all participants.

### Measures

Participants' information about social-demographics, mental health, clinical examinations, and treatments was included, providing adequate information of a T2DM patient. Reliable data from the hospital was used to build CVD risk prediction models in this study, which rendered the adoption of the prevention model in clinical practice feasible.

In this study, socio-demographic information included age, sex, marital status, educational level, income, smoking, and drinking habits. Clinical indicators were assessed during clinical examination in the hospital, including hemoglobin A1c (HbA1c), fasting glucose, body mass index (BMI), systolic blood pressure (SBP) and diastolic blood pressure (DBP), TC, HDL-C and LDL-C, triglycerides, diabetes duration, and insulin treatment condition.

Self-Rating Anxiety Scale, created by William W.K. Zung in 1971, was used to measure the anxiety symptoms of the patient with T2DM (Zung, 1971). There are 20 items, which are evaluated by 1–4 score. The raw sum score of the scale ranges from 20 to 80, but results are usually presented as the Self-Rating Anxiety Index, which is obtained by expressing the raw score converted to 100 points scale. Higher index scores on the Self-Rating Anxiety Scale reflected increasing levels of anxiety symptoms.

Self-Rating Depression Scale, created by William W.K. Zung in 1965, was used to measure the depressive symptoms of the patient with T2DM (Zung, 1965). There are 20 items, which are evaluated by 1–4 score. The raw sum score of the scale ranges from 20 to 80, but results are usually presented as the Self-Rating Depression Index, which is obtained by expressing the raw score converted to 100 points scale. Higher index scores on the Self-Rating Depression Scale reflected increasing levels of depression symptoms.

### Statistical Analysis

All analyses were performed in R (version 4.0.2). All tests were two-tailed, with a statistical significance level set at *p* < 0.05. The Mann-Whitney *U*-test, *t*-test, and Chi-Square tests were performed to compare the differences in bio-psycho-social characteristics between patients with CVD and those without CVD.

This study applied Deep Neural Network (DNN) to develop a CVD risk prediction model in patients with T2DM. DNN is typically used to make a prediction or classification through a series of layers, each of which combines an affine operation and a non-linearity. The DNN consists of an input layer, an output layer, and several hidden layers. In the structure, each layer receives the output of the previous layer as its input and passes the output to the next layer. Back-propagation is the dominant algorithm used in training neural networks. For CVD classification, participants were labeled as having the disease (label = 1) and not having the disease (label = 0) according to clinical diagnosis. We first pre-processed data. Then, the dataset was statistically analyzed and randomly split into a 75% train set and a 25% test set. In the training phase of the model development, the train dataset was used to generate a learned model prediction. In the validation phase, the model was tested with the input variables of the test dataset to evaluate how well they predicted the corresponding class labels of the test dataset. Ten-fold cross-validation was used to assess predictive performance and general error estimates in the machine learning process. Finally, the performance of CVD risk prediction model generated through learning was evaluated by the receiver operating characteristic curve (ROC) analysis. Area Under Curve (AUC), the classification accuracy, sensitivity, and specificity were used to evaluate model performance. An AUC of 1 indicates perfect discrimination ability, while an AUC of 0.5 provides worthless performance. Accuracy corresponds to the percentage of correctly predicted outcomes. Sensitivity reflects the model's ability to correctly identify the positive incidents, while specificity represents the percentage of the correctly predicted negative for CVD outcomes.

Additionally, mean impact value (MIV) is one of the best indexes to evaluate the effect of the variable on the results in the neural network application (Jiang et al., 2013). After building the DNN model, we got two new train datasets in which every independent variable increased and decreased 10%, respectively. The two datasets were used for simulation according to the fitting model. MIV refers to the mean of the difference of these two simulation results calculated by sample size. In this study, we used MIV to select the independent variables which had a great impact on the CVD risk prediction model.

## Results

### Sample Characteristics

In this study, a total of 834 questionnaires were collected. Of these patients with T2DM, 272 (32.6%) were diagnosed with cardiovascular complications. The average age of participants was 52.58 ± 11.53 years, with a range of 25–78 years. Of these patients, 489 (58.6%) were males and 345 (41.4%) were females. Among these participants, there were 156 (18.7%) smokers and 244 (29.3%) drinkers.

As Table 1 showed, there were significant differences in the comparison of sex, age, educational level, HbA1c, fasting glucose, TC, HDL-C, LDL-C, triglycerides, SBP, DBP, BMI, anxiety, depression, smoking, drinking, diabetes duration and insulin treatment condition between patients with CVD and those without CVD.

**Table 1:** Characteristics comparison between with CVD patients and without CVD patients (*N* = 834).

**Characteristics**	**Without CVD *N*(%) or M(SD)**	**With CVD *N*(%) or M(SD)**	***P***
**Sex**			0.013
Male	346(41.5)	143(17.1)	
Female	216(25.9)	129(15.5)	
**Age (years)**	46.42(10.85)	59.33(6.00)	0.000
**Marital status**			0.204
Married	515(61.8)	256(30.7)	
Single	47(5.6)	16(1.9)	
**Educational level**			0.016
Primary school education	43(5.2)	39(4.6)	
Junior high school education	157(18.8)	74(8.9)	
Senior high school education	164(19.7)	81(9.7)	
Undergraduate education	182(21.8)	75(9.0)	
Postgraduate education	16(1.9)	3(0.4)	
**Income (Yuan/month)**			0.895
≤ 1,000	77(9.2)	35(4.2)	
1,000–2,000	118(14.1)	63(7.6)	
2,000–5,000	235(28.2)	115(13.8)	
≥5,000	131(15.7)	58(7.0)	
Others	1(0.1)	1(0.1)	
**Diabetes duration (years)**	1.35(1.76)	6.56(3.93)	0.000
**Insulin treatment**			0.004
Yes	233(27.9)	85(10.2)	
No	329(39.5)	187(22.4)	
**HbA1c**	7.48(2.98)	10.03(1.41)	0.000
**Fasting Glucose**	8.28(2.00)	10.26(2.08)	0.000
**TC**	4.25(0.87)	5.31(0.93)	0.000
**HDL-C**	1.49(0.52)	1.07(0.21)	0.000
**LDL-C**	2.23(0.90)	3.31(0.80)	0.000
**Triglycerides**	2.17(1.86)	3.50(1.58)	0.000
**SBP**	125.06(13.35)	143.86(8.11)	0.000
**DBP**	80.72(8.92)	87.93(6.11)	0.000
**BMI (kg/m**^**2**^**)**	24.67(3.29)	26.97(2.52)	0.000
**Anxiety**	45.71(5.89)	76.10(8.72)	0.000
**Depression**	48.79(4.98)	81.93(5.93)	0.000
**Smoking**			0.001
Yes	87(10.4)	69(8.3)	
No	475(57.0)	203(24.3)	
**Drinking**			0.012
Yes	149(17.9)	95(11.4)	
No	413(49.5)	177(21.2)	

### CVD Risk Prediction Model in Patients With T2DM

**Figure 1:**
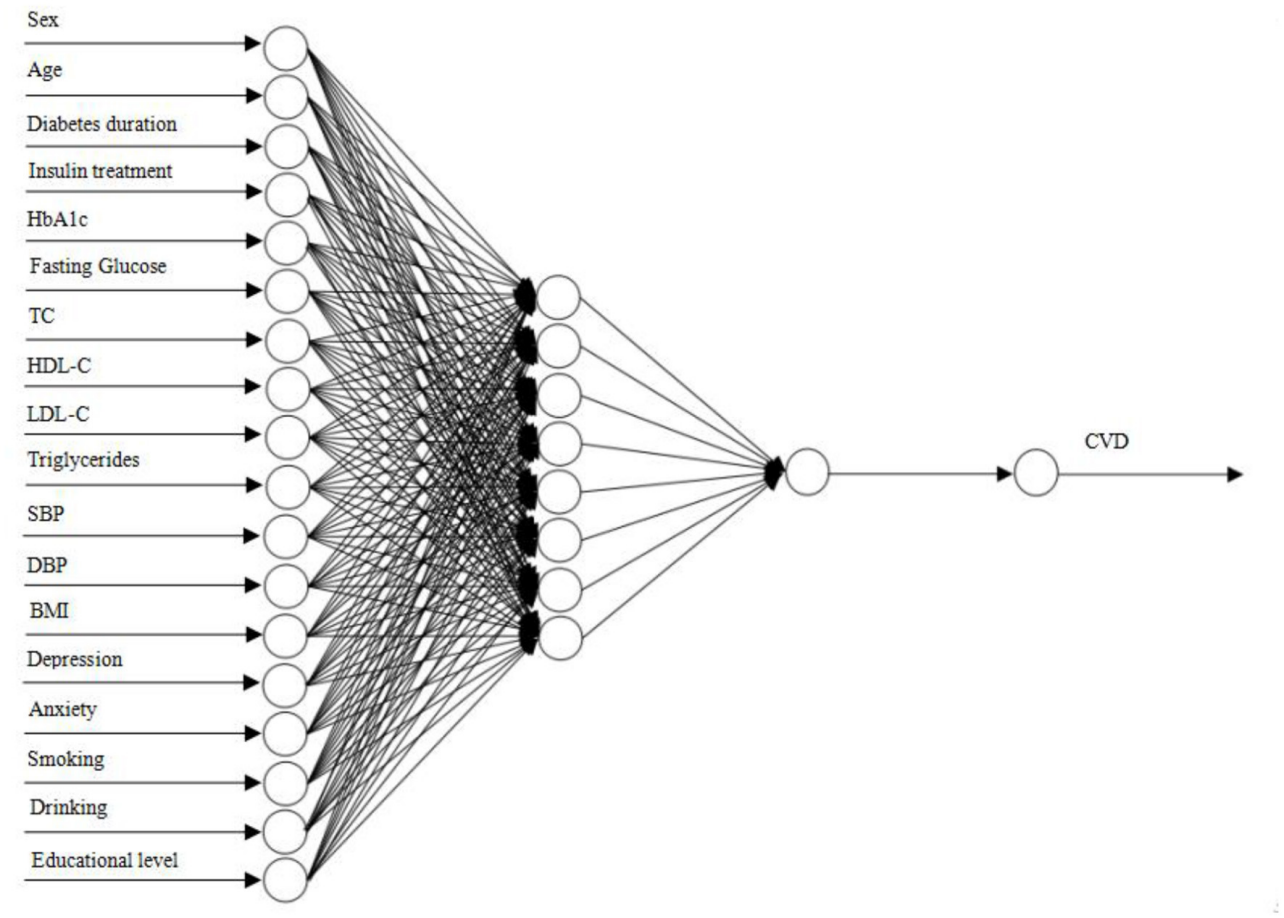
Deep neural network.

**Figure 2:**
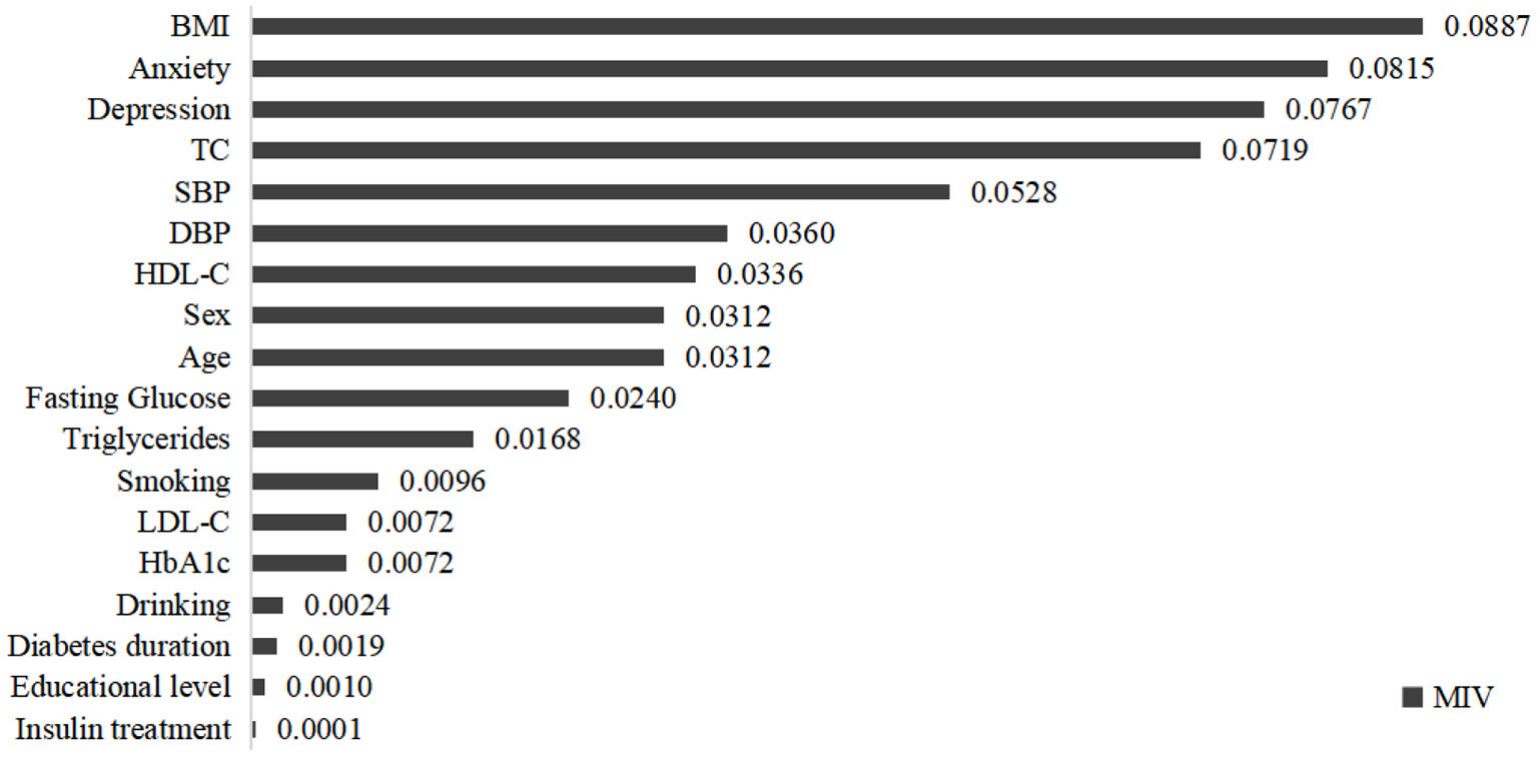
The mean impact values of independent variables in CVD risk prediction model.

In this study, a DNN with 18-8-1-1 network framework was constructed (Figure 1). The input of DNN model was all independent variables after min-max normalization. According to ROC analysis, the AUC was 0.91 in the performance evaluation of the DNN model. Table 2 showed the confusion matrix. The accuracy, sensitivity and specificity of the CVD prediction model were 87.50, 88.06, and 87.23%, respectively. In addition, the MIVs of independent variables were present in Figure 2. Among T2DM patients, the top five predictors in the CVD risk model were BMI, anxiety, depression, TC and SBP.

**Table 2:** Confusion matrix.

**Classes**		**Actual class**	
		**Negative**	**Positive**
**Predicted class**	**Negative**	123	8
	**Positive**	18	59

*Accuracy = (TP + TN)/(TP + FN+FP + TN); Sensitivity (TP rate) = TP/(TP + FN); Specificity (TN rate) = TN/(FP + TN)*.

## Discussion

The estimation of cardiovascular complications risk is essential in diabetes medical care. Guidelines for the management of T2DM advocate calculating the CVD risk to initiate appropriate treatment. In this study, we developed a CVD risk prediction model by using a machine learning method and identified key variables within the data contributing to CVD risk among T2DM patients. Results showed that the developed ensemble model for CVD risk achieved an AUC score of 0.91, accuracy of 87.50%, sensitivity of 88.06%, and specificity of 87.23%. Biological, psychological, and social factors play important roles in the progression of CVD. Among T2DM patients, the top five predictors in CVD risk model were BMI, anxiety, depression, TC, and SBP in this study.

Consistent with previous studies (Kothari et al., 2002; Mahmood et al., 2014; Lee et al., 2019), clinical indicators were the main indicators of CVD risk, especially TC and SBP. In addition, the results of this study indicated that BMI was the strongest predictor of the incidence of cardiovascular complications. In China, a paradigm shift in socioeconomic and demographic status leads to rapid urbanization and the change of the population's diet and lifestyle. This has increased the availability of unhealthy diets with high-calorie content, such as large amounts of processed meat, highly refined carbohydrates, sugary beverages, and unhealthy fats (Ley et al., 2014). T2DM continues to become more and more prevalent owing to the change in lifestyle and diet preference besides genetic predisposition. Patients with T2DM generally show a preference for an unhealthy high-calorie diet, sedentary habits, and less exercise, contributing to elevated BMI or obesity, as well as a high level of TC. Among patients with T2DM, elevated BMI was associated with a progressively higher risk of adverse cardiovascular outcomes (Gray et al., 2015). Miras et al. revealed that there was a significant improvement in many cardio-metabolic factors among T2DM patients after obesity surgery (Miras et al., 2019). Additionally, the Framingham Heart Study has identified that high blood pressure and high cholesterol levels were independent key risk factors of heart diseases (Andersson et al., 2019). Consistent results were obtained in this study. With a machine learning method, this study showed that high levels of SBP and TC were two of the top five contributors to the CVD risk prediction model. High blood pressure is highly prevalent among diabetic patients owing to the long-term hyperglycemia symptoms and metabolic syndrome (Libianto et al., 2018). A high level of SBP has been recognized as the single greatest CVD risk factor worldwide. Particularly in T2DM patients, strict blood pressure management is extremely necessary. In addition to SBP, a high level of TC is another major predictor of cardiovascular risk (Zhang et al., 2019). Patients with T2DM show a cluster of lipid abnormalities which is related to cholesterol and fatty acid accumulation in pancreatic β-cells (Perego et al., 2019). Altered regulation of the synthesis, absorption and excretion of cholesterol may contribute to atherosclerosis (Wang et al., 2017). High levels of TC and LDL-C are important risk factors for CVD, while HDL-C helps to reduce CVD risk. In this study, the rankings of TC, HDL-C, and LDL-C were 4th, 7th, and 13th, respectively. In clinical practice, DM patients would be treated with hypolipidemic drugs to correct the atherogenic dyslipidemia characteristic when necessary (Perego et al., 2019).

Particularly, although previous studies have confirmed the role of psychological determinants in the prognosis of chronic diseases (Rutledge et al., 2009; Sibelli et al., 2016; Allabadi et al., 2019), few studies have incorporated it into CVD risk prediction models. Anxiety and depression, the most common mental disorders, were included in the input variables of the CVD prediction model in the current study. The most surprising finding was that anxiety and depression had great impacts on the CVD risk of T2DM patients, ranking 2nd and 3rd, respectively.

Chronic disease management brings a great burden to patients and leads to a variety of mental disorders. Depression is a common and serious mental disorder that negatively affects emotional and cognitive functions, leading to feelings of sadness, loss of interest, and even self-injury behaviors (Tonhajzerova et al., 2020). Anxiety is characterized by transient fear, uncertainty, and apprehension about the future (Emdin et al., 2016). According to the emotional triggering model, mental stress and emotional arousal can act as triggers of cardiac dysfunction or myocardial ischemia underpinning the negative mind-heart interplay (Jiang, 2015). Physiologic changes may play a major role in the links between mental disorders and adverse CVD outcomes. Changes in the hypothalamic-pituitary-adrenal axis, the sympathetic nervous system, inflammatory processes, endothelial damage, and impaired coronary flow reserve ultimately lead to atherosclerosis, coronary artery disease, and acute coronary events (Pedersen et al., 2017). Both anxiety and depression contribute to baroreflex dysfunction, impaired endothelial function, reduced heart rate variability, arrhythmias, and then increase the risk of cardiovascular complications among patients with T2DM (Celano et al., 2018). In addition to physiologic mechanisms, behavioral mechanisms may also help to explain the associations between mental disorders and CVD risk. The negative psychological status may make it more challenging for patients with T2DM to maintain a healthy diet, exercise regularly, adhere to medications, reduce drinking, or stop smoking (Johnson et al., 2016). Given the strong effects of mental disorders on CVD risk, it is necessary for healthcare workers to pay more attention to the mental health of patients with T2DM. Effective treatment measures should be taken in standard clinical care for cardiovascular complications prevention, such as cognitive-behavioral treatment, smoking cessation, and alcohol treatment.

To fight the health and economic burden of cardiovascular complications in patients with T2DM, increasing effort to modify adverse mind-heart interactions is urgently needed. In addition to traditional risk factors for CVD, psychological determinants are now regarded as important factors that influence health in general and particularly cardio-metabolic pathways. Integrated treatment measures should be taken in the management of T2DM, including clinical care, mental health improvement, and health behavior promotion.

## Limitations

This article has several notable limitations. First, the study participants were recruited from one hospital in Harbin, leading to a potential sampling bias. Consequently, future studies should examine whether this finding can be replicated in other samples, including samples drawn from other hospitals and community populations. Second, since the research is cross-sectional, we cannot draw the conclusion that the associations between factors are truly causative. Third, the application of self-rating scales for the assessment of anxiety and depression symptoms represents a limitation of the study because of the possible alexithymia traits of TD2M patients, which can lead to an underestimation of the values of these two mental disorders (Martino et al., 2020). Moreover, given a limited number of input variables in our CVD risk prediction model, more bio-psycho-social predictors should be included in the model building in future studies.

## Conclusion

CVD was highly prevalent (32.6%) in patients with T2DM. Machine learning models can provide an automated identification mechanism for patients at risk for CVD. In addition to traditional risk factors, psychological determinants play essential roles in the CVD risk prediction model. Integrated treatment measures should be taken in the management of T2DM, including clinical care, mental health improvement, and health behavior promotion.

## Data Availability Statement

The raw data supporting the conclusions of this article will be made available by the authors, without undue reservation.

## Ethics Statement

The studies involving human participants were reviewed and approved by Ethics Committee of Harbin Medical University. The patients/participants provided their written informed consent to participate in this study.

## Author Contributions

HC: conceptualization, formal analysis, methodology, software, visualization, and writing - original draft. HP and LC: conceptualization. XY, XQ, XS, EZ, and JZ: investigation. ZQ: data curation and supervision. WZ and AM: literature research. YY: conceptualization, project administration, resources, and validation. All authors contributed to the article and approved the submitted version.

## Conflict of Interest

The authors declare that the research was conducted in the absence of any commercial or financial relationships that could be construed as a potential conflict of interest.

## Abbreviations

DM: diabetes mellitus
T2DM: type 2 diabetes mellitus
CVD: cardiovascular disease
TC: total cholesterol
HDL-C: high-density lipoprotein cholesterol
LDL-C: low-density lipoprotein cholesterol
HbA1c: hemoglobin A1c
BMI: body mass index
SBP: systolic blood pressure
DBP: diastolic blood pressure
ROC: receiver operating characteristic curve
AUC: area under curve
MIV: mean impact value.
